# The arsenic hyperaccumulating *Pteris vittata* expresses two arsenate reductases

**DOI:** 10.1038/srep14525

**Published:** 2015-09-28

**Authors:** Patrizia Cesaro, Chiara Cattaneo, Elisa Bona, Graziella Berta, Maria Cavaletto

**Affiliations:** 1 Università del Piemonte Orientale, DiSIT- Dipartimento di Scienze e Innovazione Tecnologica, viale T. Michel 11, 15121 - Alessandria, Novara, Vercelli - Italy

## Abstract

Enzymatic reduction of arsenate to arsenite is the first known step in arsenate metabolism in all organisms. Although the presence of one mRNA arsenate reductase (*PvACR2*) has been characterized in gametophytes of *P. vittata,* no arsenate reductase protein has been directly observed in this arsenic hyperaccumulating fern, yet. In order to assess the possible presence of arsenate reductase in *P. vittata*, two recombinant proteins, ACR2-His6 and Trx-His6-S-Pv2.5–8 were prepared in *Escherichia coli*, purified and used to produce polyclonal antibodies. The presence of these two enzymes was evaluated by qRT-PCR, immunoblotting and direct MS analysis. Enzymatic activity was detected in crude extracts. For the first time we detected and identified two arsenate reductase proteins (PvACR2 and Pv2.5–8) in sporophytes and gametophytes of *P. vittata*. Despite an increase of the mRNA levels for both proteins in roots, no difference was observed at the protein level after arsenic treatment. Overall, our data demonstrate the constitutive protein expression of PvACR2 and Pv2.5–8 in *P. vittata* tissues and propose their specific role in the complex metabolic network of arsenic reduction.

Arsenic (As) is a metalloid naturally occurring in the earth’s crust and released in the environment as consequence of erosion, volcanic emissions, etc. Soils can be highly contaminated by As coming mainly from mining and smelting activities, coal combustion and the use of As-containing agrochemicals^1^. Then As can spread in soil and diffuse in groundwater, entering the food chain through drinking water and contaminated vegetables^2^. This metalloid is highly toxic to most biological systems and it has been recognized as carcinogenic for human by IARC (International Agency for Research on Cancer)^3^.

Plant sensitivity to As varies according to species and a relatively low number of plant species are naturally tolerant to As; among them, *Pteris vittata* and other members of the Pteridaceae are able to hyperaccumulate As without showing any symptom^4^,^5^,^6^,^7^.

*P. vittata* L. (Chinese brake fern) is the first discovered As-hyperaccumulating plant^4^. This fern is able to remove large amounts of As from soil and shows interesting growth characteristics, including large biomass, extensive root system, high growth rate and perennial habit. *P. vittata* mostly concentrates As in fronds which is a typical feature of hyperaccumulators^4^,^8^ even though As concentration in roots can reach 100 mg kg^−1^ 9. Such As concentration can be considered very toxic for the majority of plants, but not for *P. vittata* which can tolerate up to 10,000 mg kg^−1^ 10.

In the environment, As can exist as inorganic or organic species, but it occurs predominantly in inorganic forms. The arsenate (AsV), the highest oxidized form, and the arsenite (AsIII), the highest reduced form, are predominant in aerobic and anaerobic environments, respectively^2^. The organic species of AsV that are found at low concentrations in most soils include monomethylarsonicacid, dimethylarsinicacid and trimethylarsineoxide^2^.

In plants exposed to As, AsV is readily reduced, both enzymatically and non- enzymatically, to AsIII. AsV can be directly reduced to AsIII by arsenate reductase (ACR), an enzyme first isolated from bacteria and yeasts^11^. Despite their common function, the arsenate reductases identified up to now are members of several independent families that have convergently evolved in prokaryotes and eukaryotes. The *Saccharomyces cerevisiae* arsenate reductase ACR2p^12^,^13^,^14^ belongs to the enzyme family that uses glutaredoxin (GRX) as hydrogen donor. It is homologous to the CDC25A cell-cycle protein phosphotyrosine phosphatase (PTPase)^15^ and rhodanese, a thiosulphate transferase^16^. Using functional complementation by phenotypic suppression screening of a *P. vittata* cDNA library in yeast, a *PvACR2* arsenate reductase gene was identified in the fern *P. vittata*^5^. Hereafter ACR activity have been detected in the recombinant proteins of *Arabidopsis thaliana* (AtAsr/AtACR2)^17^, *P. vittata* (PvACR2)^5^, and *Oryza sativa* (OsACR2.1 and OsACR2.2)^18^. Very recently a novel arsenate reductase gene *HAC1*/*ATQ1* has been identified in *A. thaliana* by genome-wide association mapping for the identification of quantitative trait loci related with arsenic accumulation and tolerance^19^,^20^. AtACR2 shows phosphatase activity, while the PvACR2 enzyme, like ScAcr2 protein, does not^5^,^18^,^21^. Also like ScAcr2p, the PvACR2 enzyme uses glutathione (GSH) and GRX as electron sources^5^,^18^, suggesting that the catalytic cycle involves the formation of a mixed disulfide between GSH and ACR2 that is resolved by GRX^11^. As a result of this activity, which is considered as the first step in the major As detoxification pathways found in plants^22^,^23^, more than 90% of the As in the root and in the shoot is turned into arsenite (AsIII)^22^,^24^,^25^,^26^.

Moreover, AsV is an analogue of inorganic phosphate (Pi) and is easily transported across the plasmalemma by Pi transporters (PHT)^27^. This competition between AsV and Pi for the same transport systems has been observed in As hyperaccumulators^28^,^29^, As-tolerant non-hyperaccumulators^30^ and As-sensitive non-accumulators^31^,^32^.

Although the presence and the expression level of one arsenate reductase gene (*PvACR2*) has been characterized in gametophytes^5^, till now no arsenate reductase protein has been directly observed in *P. vittata*.

The aim of this work was to characterize two arsenate reductase proteins, PvACR2 and Pv2.5–8 (gi: 89243488) in both gametophytes and sporophytes of *P. vittata* in order to improve the current knowledge about the metabolic pathways of As detoxification occurring in this plant.

## Results

In order to evaluate the impact of a chronic exposure to arsenic, ferns were treated, once a week, for 60 days with 334 μM As, which is a non-lethal dose for *P. vittata*. Arsenic did not affect frond development. On the contrary, root dry biomass was reduced (−42%) in As-treated plants (Supplementary Table S1, online). The development of gametophytes was inhibited in presence of high As concentration (8 mM) (Supplementary Table S1, online).

**Figure 1:**
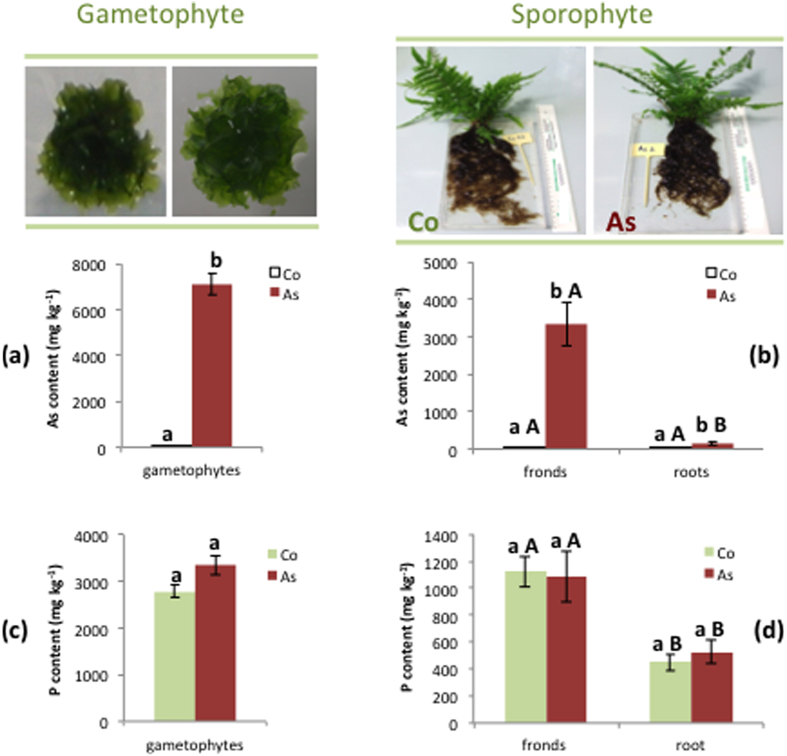
As and phosphorus concentration in *P. vittata* tissues. Each value represents the mean of five replicates (n = 5) and its standard errors (±SE). Values followed by the same letter are not different, according to Fisher’s least significant difference test at a p ≤ 0.05. Capital letters and small letters indicated a comparison between different organs or treatments, respectively.

*P. vittata* plants hyperaccumulated As in fronds (Fig. 1b), in accordance with literature^6^. In particular, 60 days after As treatment the metalloid concentration in fronds (3350.00 ± 597.24 mg kg^−1^) was 21 times higher than in roots (157.14 ± 51.68 mg kg^−1^). Although being higher in fronds than in roots, the P content inside these fern tissues was unaffected by As exposure (Fig. 1). A significant increase of arsenic content was observed in As treated gametophytes (7106.67 ± 454.67 mg kg^−1^) (Fig. 1) while the phosphorus concentrations was unaffected by As exposure (Fig. 1).

**Figure 2:**
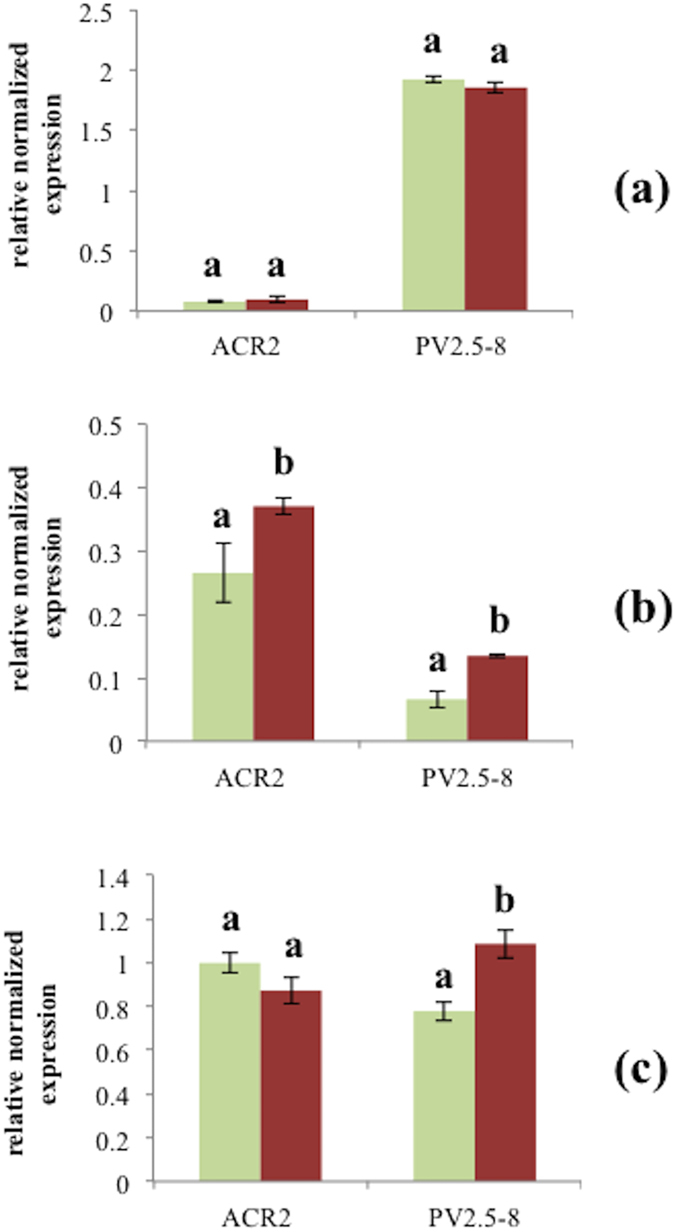
mRNA expression levels of *PvACR2* and *Pv2.5–8* in *P. vittata* fronds (a), roots (b) and gametophytes (c) by qRT-PCR. Green columns indicate controls whereas brown columns indicate As treatment. Each value represents the mean of three qRT-PCR replicates (n = 3) and its standard errors (±SE) repeated at least three times for each cDNA, from three different RNA extractions. Values followed by the same letter are not different, according to Fisher’s least significant difference test at a p ≤ 0.05.

In order to evaluate *PvACR2* and *Pv2.5–8* expression profiling in fronds, roots and gametophytes of *P. vittata* in response to As treatment, quantitative RT-PCR (qRT-PCR) (Fig. 2) was performed using *EF-1b* to compare the level of expression of *PvACR2* and *Pv2.5–8* genes. In qRT-PCR analysis, the mRNA level of *PvACR2* gene in gametophytes and sporophyte fronds exposed or not to As was not altered, also *Pv2.5–8* gene expression in sporophyte fronds was not affected by As treatment (Fig. 2a,c). On the contrary, the expression of the two genes in sporophyte roots and the *Pv2.5–8* gene in gametophytes were boosted by As treatment (Fig. 2b,c).

In order to assess the presence of arsenate reductase activity in fronds, roots and gametophytes of *P. vittata*, soluble proteins were extracted in mild conditions. Arsenate reductase activity was monitored by the coupled assay described by Ellis *et al.*^5^ with minor modifications. Arsenate reduction is coupled to NADPH oxidation via the reduction of oxidized GSH by GR, with GSH serving as the electron donor for arsenate reduction. Rate of NADPH oxidation was measured as decrease of optical density at 340 nm and was found to be minimal in the absence of frond, root and gametophytes protein extracts. Enzyme assay in the absence of arsenate was also performed as negative control. The decrease of NADPH absorbance at 340 nm observed in presence of the protein extract and the enzyme substrate (40 mM arsenate) was deducted by the absorbance reduction in presence of protein extract without arsenate. The contribution of the NADPH self-oxidation was valued by calculating the difference between the absorbance (∆Abs) observed in the mixture assay without the protein extract, and the ∆Abs observed in presence of the protein extract. Arsenate reductase activity in fronds was inhibited by As exposure (Table 1), while in roots and gametophytes was unaffected by As treatment; in accordance with Ellis *et al.*^5^ , that reported a constitutive arsenate reductase activity in gametophytes.

*P. vittata* sporophytes and gametophytes grown with or without As were used for the detection of PvACR2 and Pv2.5–8 in fern tissues using polyclonal antibodies. In particular, two recombinant proteins containing His6-tags, ACR2-His6 and Trx-His6-S-Pv2.5–8 were prepared in *E. coli*, purified under denaturing conditions, and used as antigens in rabbit. The ability to identify PvACR2 and Pv2.5–8 and the title of polyclonal antibody products were compared with commercial monoclonal antibody anti-His_6_ tag signal (data not shown).

**Figure 3:**
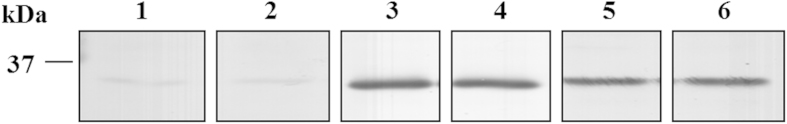
Immunoblot with anti-Pv2.5–8 antibody on *P. vittata* roots (lanes 1–2), fronds (lanes 3–4) treated or not once a week with 334 μM and gametophytes treated or not with 8 mM As (lanes 5–6). Controls: lanes 1, 3, 5; arsenic treatment: lanes 2, 4, 6.

**Figure 4:**
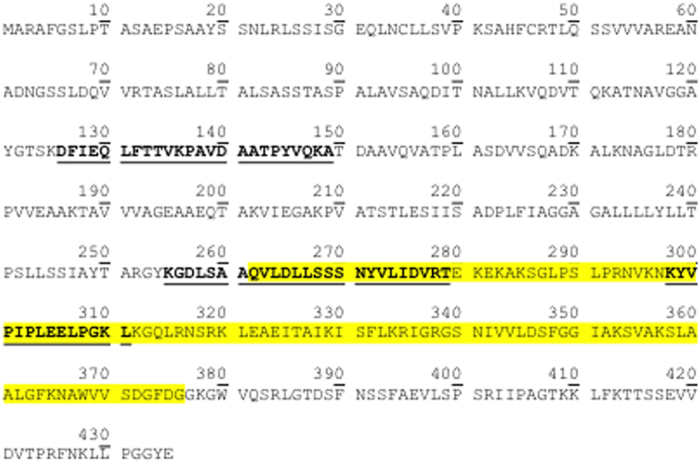
Pv2.5–8 amino acidic sequence (data from NCBI database): 435 amino acids and theoretical pI 9.31. The Rhodanese homology domain (262–376) is yellow highlighted. The peptides identified by nanoLC-MS/MS analysis are in bold and underlined.

No bands were detected for PvACR2 protein in any of the fern tissues (data not shown), whereas anti-Pv2.5–8 antibodies detected a protein band in fronds, gametophytes and, to a minor extent, in roots (Fig. 3). The MW of this protein band was about 34.55 kDa, which is lower than the theoretical MW of Pv2.5–8 (45 kDa) (gi: 89243488), whose sequence is shown in Fig. 4. The signal intensity of the protein bands did not change with arsenic treatment in any of the tested tissues. Moreover, fronds and gametophytes showed similar signal intensity, and it was higher than that observed in roots (Fig. 3).

**Figure 5:**
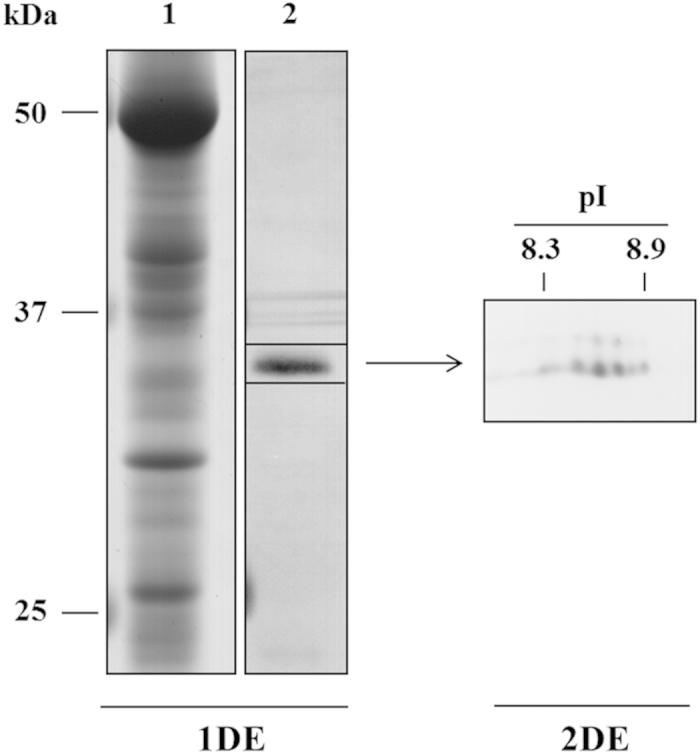
*P. vittata* sporophytes frond proteins from control, stained with Blue-Silver (lane 1), immunodetected with anti-Pv2.5–8 polyclonal antibodies in 1-DE (lane 2) and 2-DE (panel).

In order to characterize this 34 kDa protein , the gel portion corresponding to the 25–50 kDa region, was transferred onto PVDF membranes, probed with anti-Pv2.5–8 polyclonal antibodies, excised, digested with trypsin and finally submitted to nanoLC-MS/MS analysis (Fig. 5). Four peptides corresponding to the Pv2.5–8 putative arsenate reductase were successfully identified and three of them partly cover the rhodanese homology domain (from aa 262 to aa 376) of this protein (gi: 89243488) (Fig. 4, Table 2). Then, to further characterize Pv2.5–8, 2-DE was performed. Interestingly, in fronds and gametophytes, Pv2.5–8 migrates as a series of spots having the same MW (about 35 kDa) of the protein band isolated on 1-DE gels, but with different isoelectric point (pI). More in detail, six spots with pI ranging from 8.3 to 8.9 were detected (Fig. 5). Fronds and gametophytes showed a similar protein pattern, regardless As treatment (data not shown). 2-DE was performed on root extracts too, but no spots were detected by antibodies.

**Figure 6:**
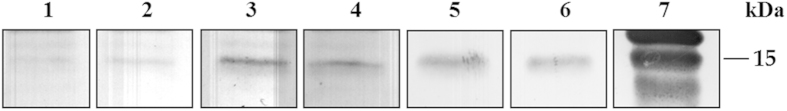
Immunoblot after immunoprecipitation with anti-ACR2 antibody from roots (lanes 1–2) and fronds (lanes 3–4) of *P. vittata* sporophytes treated or not with 334 μM As, and gametophytes (lanes 5–6) treated or not with 8 mM As. Controls: lanes 1, 3, 5; arsenic treatment: lanes 2, 4, 6. Recombinant ACR2 (lane 7) was used as a positive control of immunoprecipitation.

In order to detect PvACR2 in fern tissues, gametophyte and frond/root extracts were immuno-precipitated with anti-ACR2 polyclonal antibodies (Fig. 6). In all the immune-precipitated tissues anti-ACR2 polyclonal antibodies detected a band of about 14.4 kDa and this protein is present to a minor extent in roots, as observed for Pv2.5–8. This protein showed the same MW of the ACR2 recombinant protein.

## Discussion

It’s well known that in As hyperaccumulator plants, such as *P. vittata*, arsenate (AsV) can be directly reduced to AsIII by arsenate reductase^24^. However, the plant organ where the AsV reduction occurs in *P. vittata* is still unknown. Some authors^33^,^34^ suggested that AsV is reduced to AsIII in the roots, and subsequently transported to the fronds. Other works^6^,^35^ reported that AsV reduction to AsIII takes place directly in the fronds. More recently, AsV reduction has been observed in the rhizomes and in the pinnae but not in the roots of this fern^36^.

In our experimental conditions, sporophytes and gametophytes of *P. vittata* did not show macroscopic stress symptoms showing a more high tolerance of this fern to the metalloid. Arsenic treatment reduced the dry weight of the gametophytes and roots compared to controls. Consistently with previous works^8^,^37^ we found a higher amount of As in gametophytes and fronds of *P. vittata*. It has been postulated that in As hyperaccumulating plant species, As is not immobilized in roots, but it is transported through the xylem to the fronds^6^,^38^ and sequestered as free AsIII in the vacuole^6^,^38^. As recently demonstrated by Indriolo *et al.*^39^, the PvACR3 protein is involved in the vacuolar sequestration of AsIII in *P. vittata*.

Since AsV is a chemical analogue of phosphate, arsenic uptake by *P. vittata* occurs through phosphorus transporters^28^. Therefore, a competition between AsV and phosphate for the same transporter is expected to occur in plants grown in As polluted soils. However, according to Bona *et al.*^8^ that observed no differences in the roots, our results showed that the phosphorus content inside the plant organs was unaffected by As exposure, despite a high variability of the P content in each plant.

The *PvACR2* mRNA levels were unaffected by exposure to different arsenic amounts (334 μM and 8 mM, respectively) in fronds and gametophytes of *P. vittata*. On the contrary, *Pv2.5–8* mRNA levels increased in As-treated gametophytes, moreover an increase of both *PvACR2* and *Pv2.5–8* mRNA levels occurred in arsenic treated roots. The constitutive expression of *PvACR2* in gametophytes of *P. vittata* is consistent with the findings of Ellis *et al.*^5^. However, the presence of *Pv2.5–8* and *PvACR2* mRNAs in fronds and roots, and of *Pv2.5–8* in gametophytes has never been reported before. These data suggest the involvement of these two proteins in the plant response to arsenic and can support, on the basis of mRNA levels up-regulation, the hypothesis that AsV reduction to AsIII occurs mainly in roots.

In a further experimental step, we detected and identified for the first time the two arsenate reductases (Pv2.5–8 and PvACR2) in both sporophytes and gametophytes of *P. vittata* at the protein level. The isolation of *PvACR2* and *Pv2.5–8* cDNAs from *P. vittata* tissues revealed the presence of *PvACR2* in sporophytes, and that of *Pv2.5–8* in both gametophytes and sporophytes. Pv2.5–8 corresponds to a “predicted protein sequence of a putative arsenate reductase” submitted in 2006 to GenBank (Pv2.5–8, gi: 89243488) as an aldose reductase and rhodanese, similar to calcium binding protein (Rathinasabapathi *et al.* unpublished). The specific antibodies produced against Pv2.5–8 detected a protein band in fronds, gametophytes and, to a minor extent, in roots showing a molecular weight (about 35 kDa) lower than the theoretical MW of Pv2.5–8 (45 kDa). Since the 35 kDa protein band has been identified by MS/MS analysis as Pv2.5–8 (gi: 89243488), it’s likely that post-translational cleavage, not predictable at the transcription level, have occurred in fern tissues. More in detail, three of the identified peptides were part of the Rhodanese Homology Domain of this protein, suggesting that the catalytic portion of the protein remains unaltered, while a protein cleavage may occur in the N-terminal side of the amino acid sequence. Moreover, the detection of Pv2.5–8 on 2-DE gels using anti-Pv2.5–8 antibody as series of spots with the same molecular weight (35 kDa), but with different pIs, could be related to post-translational modifications, such as phosphorylation, where the addition of one or more phosphate group alters the protein pI leading to its acidification. The signal intensity of Pv2.5–8 in fronds and gametophytes was unaffected by As treatment. Moreover, the signal intensity of Pv2.5–8 in roots was lower than that detected in fronds and gametophytes. Despite the *Pv2.5–8* mRNA levels in roots and gametophytes increased after As treatment, the protein Pv2–5–8 level did not change.

The immunoprecipitation of gametophyte and frond/root protein extracts revealed in each tissue the presence of a protein band of about 14 kDa, with the same MW of PvACR2, confirming the expression of ACR2 protein in *P. vittata* gametophyte and sporophyte. It is surprising that a so relevant enzyme for the arsenic metabolism (PvACR2) is expressed in such a low amount in *P. vittata*. Its presence in fronds and gametophytes seems to be higher than in roots, as observed also for Pv2.5–8.

Despite the mRNA level of both proteins increased in roots after As treatment, no difference was observed at the protein level; this suggests the presence of other molecular mechanisms that regulate the synthesis and the activity of these proteins. Arsenate reductase activity was detected in frond, root and gametophyte extracts. Whereas the activity did not change in roots and in gametophytes following As treatment, higher AsV reductase activity was detected in frond of control plants compared to the arsenic treated ones. Multiple enzymes have been shown to exhibit AsV reductase activity^2^; these included glyceraldehyde-3-phosphate dehydrogenase, triosephosphate isomerase and phosphoglycerate kinase that in *P. vittata* fronds treated with As showed a lower protein expression level^8^ and probably a less reductase activity. The enzymatic activity and its trend in relation to arsenic exposure were consistent with the findings of Liu *et al.*^34^, who noticed a slight decrease of arsenate reductase activity in fronds along with the increase of arsenic added in soil up to 100 ppm, followed by an increase of the enzyme activity with arsenic concentrations higher than 100 ppm. This observation seems to be in contradiction with the detection of higher amounts of both Pv2.5–8 and PvACR2 proteins in fronds than in roots, even if unaffected by arsenic treatment. Moreover, an increase of *PvACR2* and *Pv2.5–8* transcripts after arsenic exposure was observed in roots. We can speculate that roots are not the organs for arsenic storage in *P. vittata*, consequently a constitutive high amount of arsenate reductase is not required, but its production together with a high protein turnover rate, is induced after arsenic exposure to allow the prompt arsenate reduction and transfer to fronds. On the contrary, fronds are the elected organs for the storage of arsenic: therefore, the maintenance of a constitutive pool of arsenate reductase in fronds is essential in case of re-oxidation of AsIII to AsV, which can occur in senescent leaves, where reductase activities usually decrease while oxidase activities increase^40^.

In conclusion, the constitutive expression of both *P. vittata* arsenate reductase proteins in fronds, roots and gametophytes, suggests that the reduction of arsenate is carried out by a system of arsenate reductases with the support of other enzymes having metabolic essential functions. These conclusions are sustained by other works showing the involvement of a cytosolic triosephosphateisomerase (TPI) in arsenate reduction in *P. vittata*^41^, the activation of multiple pathways to tolerate As^8^,^9^,^42^ and the newly identified arsenate reductase in *A. thaliana*^19^,^20^. Overall the hyperaccumulating fern *P. vittata* displays different molecular strategies for As tolerance in roots and fronds, which need to be considered for the characterization of its phytoremediation potential.

## Methods

### Plant material

*P. vittata* spores were sterilized as described by Trotta *et al.*^43^. After one month, half of the gametophytes were transferred to sterile polyethylene boxes for sporophyte production. The remaining gametophytes were transferred to 9 cm Petri dishes containing 15 ml liquid MS medium (Murashige and Skoog 1962)^44^ added with 2% sucrose, with or without arsenate 8 mM (supplied as Na_2_HAsO_4_·7H_2_O) (Sigma-Aldrich, MO, USA) and maintained for 10 days in a growth chamber with 16***/***8 h light/dark photoperiod, 150 μmol m^−2^ s^−1^ light irradiance and 24/20 °C thermoperiod. Then, the gametophytes were washed and weighed. Part of the samples was dried at 60 °C for 72 h and used for the determination of arsenic (As) and phosphorus (P) concentration, while the rest was frozen in liquid nitrogen and stored at −80 °C.

The sporophyte experiment was performed according to Bona *et al.*^8^,^9^.

### Arsenic and phosphorus concentration

The As and P concentrations were measured both in roots and in fronds. 0.5 g dry weight of each sample were digested in 6 ml of 65% nitric acid (Sigma-Aldrich) using a MARS 5 microwave oven (CEM, North Carolina, USA). Digested samples were analysed by inductively coupled plasma-optical emission (IRIS Advantage ICAP series DUO HR, Thermo Jarrell Ash, Franklin, USA) and inductively coupled plasma-MS (Plasma QUAD 3, VG Elemental Europe, Cedex, France). Certified standards of analysed metals and acid blanks were run. The As and P concentrations were expressed as mg kg^−1^.

### RNA extraction and quantitative RT-PCR

Total RNA was extracted from 500 mg of *P. vittata* fronds, roots and gametophytes, combining CTAB method^45^ and NucleoSpin RNA Plant kit (Macherey-Nagel, Düren, Germany). Briefly, 500 mg of tissue powdered in liquid nitrogen were added to 500 μl of extraction buffer (2% CTAB, 2% polyvinylpyrrolidone (PVPP) MW 4000, 100 mM Tris-HCl pH 8.0, 20 mM EDTA, 1.4 M NaCl and 2% β-mercaptoethanol). An equal volume of chloroform-isoamyl alcohol (24:1), previously heated for 10 min at 60 °C, was added to the mixture. The suspension was then centrifuged at 16000 g for 8 min at 4 °C. RNA was precipitated with isopropanol for 60 min at −20 °C and cleaned using the Nucleospin RNA plant Kit. RNA purity, quantity and integrity were assessed^46^. 1–2 μg of total RNA from *P. vittata* tissues were treated with Dnase I (Sigma-Aldrich) and reverse transcripted to cDNAs using oligo (dT)_18_ primer and RevertAid H Minus First Strand cDNA synthesis kit (Fermentas, Canada). Then 1 μl of sample was diluted in a DEPC-treated water solution containing 0.02 U μl^−1^ of Taq DNA polymerase (Finnzymes, Finland), 10× PCR buffer (containing 15 mM MgCl_2_) (Finnzymes), 500 μM of dNTPs (125 μM each dNTP) and 500 nM of the *PvACR2* or *Pv2.5–8* forward and *PvACR2* or *Pv2.5–8* reverse primers (Supplementary Table S2, online), and amplified by PCR in a thermocycler (Techne, Bibby Scientific, Italy). PCR conditions were 5 min at 94 °C, 30–33 cycles of 1 min at 94 °C, 30–33 cycles of 1 min at 55 °C, 30–33 cycles of 1 min and 30 s at 72 °C, 10 min at 72 °C. The resulting fragments were cloned into pCR4-TOPO vector using the TOPO TA Cloning Kit for Sequencing (Invitrogen, CA, USA) sequenced by BMR Genomics (Padova, Italy) using primer T3 and compared to the sequences of *PvACR2* and *Pv2.5–8* available in GenBank by using the BLASTn program^47^.

Quantitative RT-PCR (qRT-PCR) of *PvACR2* and *Pv2.5–8* was performed in a multiplex Taqman assays using *P. vittata* elongation factor-1b (*EF-1b*) as constitutive internal standard^5^. Probes dual-labeled and primer pairs (Supplementary Table S2, online) were designed using Beacon Designer v3.0 (Premier Biosoft International, Inc.). cDNA was amplified in a CFX384 Real-Time PCR (Bio-Rad) using 0.3 μM each primer, 0.1 μM each probe iQ^TM^ Multiplex Powermix (Bio-Rad) according to the manufacturer’s instructions for the triplex protocol, in a final volume of 10 μL. For all Taqman assays the thermal protocol was: 3 min at 95 °C, followed by 46 cycles of 15 s at 95 °C and 20 s at 59 °C. Relative expression data were geometrically normalized to *EF-1b*. qRT-PCR was performed on three different biological replicates and repeated at least three times for each cDNA.

### Protein expression, purification and antibody production

The *PvACR2* and *Pv2.5–8* reading frames were amplified by PCR with primers adding a NcoI and XhoI site to the 5′ and 3′ ends of the fragment, respectively (Supplementary Table S2, online). PCR conditions for both genes were 5 min at 94 °C, 30 cycles of 1 min at 94 °C, 30 cycles of 1 min at 58 °C, 30 cycles of 1 min and 30 s at 72 °C, 10 min at 72 °C. The fragments were digested with NcoI and XhoI, then the *Pv2.5–8* fragment was ligated into pET32a (Novagen, United Kingdom) in frame at 5′ with thioredoxin, S-tag, and His6-tag, creating plasmid pET-Pv2.5–8, and the *PvACR2* fragment was ligated into pET20b (Novagen) in frame at 5′ with pelB signal sequence and at 3′ with His6-tag, creating plasmid pET-PvACR2.

For protein expression, *E. coli* BL21DE3 cells (Stratagene, Canada) transformed with pET-PvACR2 or pET-Pv2.5–8 were grown in at 37 °C in Luria-Bertani medium containing 50 μg ml^−1^ ampicillin. At 0.5 of absorbance at 600 nm, isopropylthio-β-galactoside (IPTG) was added to a final concentration of 0.4 mM and the cell cultures were incubated for 4 h at 37 °C. Recombinant proteins were purified using the Ni-NTA Agarose resin (QIAGEN, Italy) under denaturing conditions. Recombinant PvACR2 and Pv2.5–8 were identified by SDS-PAGE^46^, dialyzed against 1000 volumes of 100 mM NaH_2_PO_4_, 10 mM Tris, 2.5 M urea, pH 7.4, concentrated using Microcon YM-3 (Millipore, Germany) and quantified by Bradford method^48^. Antigenic preparations were emulsified with complete Freund adjuvant (MP BioMedicals, Illkirch, France) and injected into NZW rabbits. A second booster injection was given 30 days later using polyA-polyU as adjuvant. At 2-wk intervals, bleeding of the rabbits was performed according to standard procedure^49^. Different serum dilutions were tested against recombinant proteins separated by SDS-PAGE and transferred onto a nitrocellulose membrane (0.2 μm or 0.45 μm). For immunodetection, membranes were saturated with PBS/BSA 5% and probed with anti-His6 monoclonal antibodies (GE-Healthcare, Cologno Monzese, Italy) or with the sera of rabbits immunized with the two *P. vittata* recombinant proteins. Immunodetection was performed using either chemiluminescence reaction or chromogenic reaction.

### Protein extraction, Two-dimensional gel electrophoresis (2-DE), nanoLC-MS/MS analysis

Proteins were extracted from gametophytes and sporophyte tissues according to Bestel-Corre *et al.*^50^ with some modifications, and 2-DE separated as reported in Bona *et al.*^8^,^9^.

In parallel, frond and root extracts were also separated by monodimensional SDS-PAGE (1-DE). 1-DE and 2-DE gels were stained with Blue Silver colloidal Coomassie, according to Candiano *et al.*^51^, or transferred onto nitrocellulose or PVDF membranes. The development of membranes from was performed as described above, using rabbit polyclonal antibodies against PvACR2 and Pv2.5–8 as the primary antibody, and an alkaline phosphatase-conjugated ‘anti-rabbit’ serum as secondary antibody (Sigma-Aldrich). The antibody reaction was detected by chromogenic reaction. Images were acquired with GS-710 densitometer (BioRad).

Nine replicates of the immuno-detected 1-DE bands were excised and trypsin digested as reported in detail in Bona *et al.*^8^. Digested samples were nanoLC-MS/MS analysed using a QSTAR XL instrument (Applied Biosystems, CA, USA) coupled with nano-flow LC system (Dionex, Amsterdam, The Netherlands). Protein identification was performed using the MASCOT search engine^52^.

### PvACR2 immunoprecipitation from plant tissues

About 600 μg of gametophyte and sporophyte protein extracts were dialysed against 5 volumes of NP-40 buffer. 1 μl of rabbit serum was added to each tube and incubated ON at 4 °C. ProteinA-Sepharose CL 4B resin (GE Healthcare) was resuspended in 1 vol of water, centrifuged at 3000 g at 4 °C for 6 min, washed twice and resuspended in water to a 1:1 ratio. 50 μl of protein A-Sepharose CL 4B were activated, added to each sample and incubated at 4 °C for 60 min. Samples were then centrifuged (3000 g, 6 min, 4 °C), the pellet was washed twice with 300 μl of PBS. The pellet was then resuspended in 40 μl of 1-DE Laemmli buffer containing β-mercaptoethanol, denaturated for 10 min at 95 °C and stored at −20 °C.

### Arsenate reductase assay of *P. vittata* extracts

Gametophytes, fronds and roots of *P. vittata* were powdered in liquid nitrogen, resuspended in five volumes of extraction buffer (50 mM MES/MOPS pH 6.5, 0.3 M KCl, 10 mM β-mercaptoethanol, protease inhibitor cocktail 1%, PVPP 0.033 g ml^−1^, glycerol 5%) and centrifuged at 1,860 g for 15 min at 4 °C. The supernatant was centrifuged at 20,000 g for 60 min at 4 °C and concentrated using Amicon Ultra-4 (Millipore) with a cutoff of 10 kDa. Arsenate reductase activity was measured using a coupled assay as described by Ellis *et al.*^5^. The assay mixture consisted of 50 mM MES/MOPS pH 6.5, 300 mM NaCl, 0.1 mg ml^−1^ BSA, 1 mM GSH, 250 μM NADPH, 1 U yeast glutathione reductase (GR), 70 μg of total protein extract, 40 mM sodium arsenate (Na_2_HAsO_4_ 7H_2_O) or an equal volume of water. The assay was carried out in a final volume of 1.5 ml at room temperature. Reductase activity was monitored for 90 min at 340 nm by a DU 800 spectrophotometer (Beckman, CA, USA). The nmoles of oxidized NADPH were calculated using a molar extinction coefficient of 6,200 M^−1^cm^−1^.

### Statistical analysis

Statistical analysis was performed with StatView 4.5 software (Abacus Concepts, CA, USA). ANOVA test, followed by a post-hoc F test with *p* ≤ 0.05 as cut off.

## Additional Information

**How to cite this article**: Cesaro, P. *et al.* The arsenic hyperaccumulating *Pteris vittata* expresses two arsenate reductases. *Sci. Rep.* **5**, 14525; doi: 10.1038/srep14525 (2015).

## Supplementary Material

Supplementary Information

## Figures and Tables

**Table 1:** Arsenate reductase activity in *P. vittata* frond and root sporophytes treated or not once a week with 334 μM As, and arsenate reductase activity in *P. vittata* gametophytes treated or not with 8 mM As.

	Frond	Root	Gametophyte
	(ncatal/mg)	(ncatal/mg)	(ncatal/mg)
**Control**	0,278 ± 0,002 a	0,177 ± 0,015 a	0,176 ± 0,030 a
**As**	0,114 ± 0,006 b	0,145 ± 0,019 a	0,154 ± 0,014 a

The enzymatic activity is estimated in ncatal/mg protein. Value followed by the same letter are not different, according to Fisher’s least significant difference test at a p ≤ 0.05.

**Table 2:** List of peptides derived from the immunoreactive (anti-Pv2.5–8) 1-DE bands and identified by MS/MS analysis.

Precursor ion m/z	Precursor ion mass	Peptide sequence	Modification	Protein	M_r_(kDa)/pI theor	AC number (gi NCBI) and reference organism	Score
677.8789	1353.7432	K.YVPIPLEELPGK.L		putative arsenate reductase	45.24/9.31	gi|89243488 *Pteris vittata*	76
861.1225	2580.3456	K.DFIEQLFTTVKPAVDAATPYVQK.A					
1224.6821	2447.3496	K.GDLSAAQVLDLLSSSNYVLIDVR.T					
1225.1615	2448.3085	K.GDLSAAQVLDLLSSSNYVLIDVR.T	Deamidated (NQ)				
