# Supplementary material for: The arsenic hyperaccumulating Pteris vittata expresses two arsenate reductases
Source: Sci Rep. 2015 Sep 28;5:14525. doi: 10.1038/srep14525 (PMC4585942; doi:10.1038/srep14525)
Supplement: Supplementary Information [file srep14525-s1.pdf]

Supplementary Information

The arsenic hyperaccumulating *Pteris vittata* expresses two arsenate reductases

Patrizia Cesaro, Chiara Cattaneo, Elisa Bona, Graziella Berta, Maria Cavaletto

Supplementary Table S1. Dry weights of *P. vittata* sporophytes (root and frond) treated or not once a week with 334 μM As, and dry weights of *P. vittata* gametophytes treated or not with 8 mM As. Each value represents the mean of five replicates (n = 5) and its standard errors (± SE). Values followed by the same letter are not different, according to Fisher's least significant difference test at a $p \leq 0.05$.

	**Control**	**As**
Root dry weight (g)	2.645 ± 0.274 a	1.542 ± 0.354 b
Frond dry weight (g)	1.400 ± 0.134 a	1.240 ± 0.255 a
Gametophyte dry weight (g)	0.020 ± 0.0002 a	0.012 ± 0.001 b

Supplementary Table S2. Primers used in this study. FAM = 6-carboxyfluorescein, HEX = hexachloro-fluoresceine, RED = Texas Red, BQ1= Black Hole Quencher 1 and BQ2 = = Black Hole Quencher 2.

**Primer**	**Modification**	**5'-3'**	**Reference**
PvACR2 for	---	ATGGCCTCGCTTCCATCC	This study
PvACR2 rev	---	TCAACACCCCTCACAAAAGG	This study
Pv2.5-8 for	---	ATGGCGAGGGCTTTTGGATC	This study
Pv2.5-8 rev	---	TTACTCGTATCCACCGGGAAG	This study
EF1b for	---	GAAGCCTTGGGATGATGAAA	Ellis et al. 2006
EF1b rev	---	CCTCGATCAGGTTGTCCACT	Ellis et al. 2006
PvACR2 NcoI for	---	ACTG<u>CCATGG</u>CCTCGCTTCCATCC	This study
PvACR2 XhoI rev	---	CAGT<u>CTCGAG</u>ACACCCCTCACAAAAGGC	This study
Pv2.5-8 NcoI for	---	ACTG<u>CCATGG</u>CGAGGGCTTTTGGATC	This study
Pv2.5-8 XhoI rev	---	CAGT<u>CTCGAG</u>TTTACTCGTATCCACCGGGAAG	This study
PvACR2 qfor	---	TCGCTTCCATCCCTCTCCTA	This study
PvACR2 qrev	---	CCAAGATCTGATCTCAGT CCTTC	This study
PvACR2 probe	5'-FAM, 3'-BHQ1	CTCTGCTACCGACCTCAT TCGTCTGC	This study
Pv2.5-8 qfor	---	CGTCGTTGGCTCTTCTCACT	This study
Pv2.5-8 qrev	---	ACCATAAGCACCTCCTACTGC	This study
Pv2.5-8 probe	5'-HEX, 3'-BHQ1	CACTCTCGGCTTCGTCTACTGCTTCTC	This study
EF1b qfor	---	AGCTTGAAGAGGCTG TTCGA	This study
EF1b qrev	---	ACAAGATCGTCTTCGATTGTCAT	This study
EF1b probe	5'-RED, 3'-BHQ2	TCAACTTCTTGATGCCGTAACCAACCG	This study
